# Binding Features and Functions of ATG3

**DOI:** 10.3389/fcell.2021.685625

**Published:** 2021-06-21

**Authors:** Dongmei Fang, Huazhong Xie, Tao Hu, Hao Shan, Min Li

**Affiliations:** School of Pharmaceutical Sciences, Sun Yat-sen University, Guangzhou, China

**Keywords:** ATG3, autophagy, binding feature, cancer, homeostasis, function, phosphatidylethanolamine, post-translational modification

## Abstract

Autophagy is an evolutionarily conserved catabolic process that is essential for maintaining cellular, tissue, and organismal homeostasis. Autophagy-related (*ATG*) genes are indispensable for autophagosome formation. *ATG3* is one of the key genes involved in autophagy, and its homologs are common in eukaryotes. During autophagy, ATG3 acts as an E2 ubiquitin-like conjugating enzyme in the ATG8 conjugation system, contributing to phagophore elongation. ATG3 has also been found to participate in many physiological and pathological processes in an autophagy-dependent manner, such as tumor occurrence and progression, ischemia–reperfusion injury, clearance of pathogens, and maintenance of organelle homeostasis. Intriguingly, a few studies have recently discovered the autophagy-independent functions of ATG3, including cell differentiation and mitosis. Here, we summarize the current knowledge of ATG3 in autophagosome formation, highlight its binding partners and binding sites, review its autophagy-dependent functions, and provide a brief introduction into its autophagy-independent functions.

## Introduction

Autophagy plays an important role in maintaining cellular energy balance, structural reconstruction, and immunity when cells respond to stress conditions, such as amino acid starvation. Based on how a cargo is transported to lysosomes, autophagy can be divided into macroautophagy, microautophagy, and molecular chaperone-mediated autophagy (Sridhar et al., 2012; Catarino et al., 2017). Macroautophagy (hereinafter referred to as autophagy) is a highly evolutionarily conserved process that sequesters cytoplasmic components, cellular organelles, invading microorganisms, and aggregated proteins into a double membrane–bound structure called the autophagosome, which then fuses with lysosomes to degrade the cargo.

The most crucial event in autophagy is autophagosome formation, which was first observed by Christian de Duve in the 1960s. A series of autophagy-related (*Atg*) genes was subsequently identified through genetic studies in yeast in the 1990s (De Duve and Wattiaux, 1966; Tsukada and Ohsumi, 1993). Currently, scientists have identified more than 40 *Atg* genes, mainly by genetic screening using model organisms, such as *Saccharomyces cerevisiae* (Morita et al., 2019). Among these *Atg* genes, one subset has been identified as the core *Atg* genes because they are required for autophagosome formation, including nucleation, elongation, and closure of the isolation membrane (Xie and Klionsky, 2007).

Two ubiquitin-like conjugation systems, the ATG12 conjugation system and Atg8/microtubule-associated protein 1 light chain 3 (LC3) lipidation system, are known to be indispensable for phagophore elongation (Shintani et al., 1999; Ichimura et al., 2000; Tanida et al., 2002a). ATG3 acts as an E2-like enzyme in the Atg8/LC3 lipidation system and is essential for the lipidation of Atg8/LC3 (Ichimura et al., 2000; Tanida et al., 2002b). Although lipidation of LC3 can occur during the non-canonical autophagic process in an ATG5/ATG7-independent manner, there is no current evidence indicating that cells deficient in ATG3 can convert LC3-I; to LC3-II (Moloughney et al., 2011; Chang et al., 2013). Although *Atg3^–/–^* mouse embryo fibroblast cells (MEFs) are survivable, *Atg3^–/–^* mice are nonviable, suggesting that Atg3 is essential for the homeostasis of the organism (Sou et al., 2008). ATG3 is conserved in eukaryotes and can interact with many proteins, such as LC3ylation (Agrotis et al., 2019). In addition, functions of ATG3 are emerging in many contexts, including the maintenance of mitochondrial homeostasis, regulation of tumor progression, and clearance of viral infection (Radoshevich et al., 2010; Altman et al., 2011; Choi et al., 2014). Recently, a few autophagy-independent functions of ATG3 were found, indicating that the roles of ATG3 might be more complex. In this review, we sought to elucidate the binding features of ATG3 in two conjugation systems and their functions in autophagy-dependent and -independent pathways.

## Roles of ATG3 in Two Conjugation Systems

Unlike ATG4, which has four subtypes, and ATG16L1, which has three subtypes, there is only one type of ATG3 in organisms. ATG3 and its homologs are common in eukaryotes, including fungi, and higher eukaryotes, such as mammals, insects, and plants. Furthermore, their amino acid sequences in different species are highly conserved (Tsukada and Ohsumi, 1993; Xu et al., 1999; Juhasz et al., 2003; Wu et al., 2006; Hanada et al., 2009).

In 1993, Ohsumi et al. first isolated 15 mutants, *apg1*–*apg15*, from *S. cerevisiae*, which could not accumulate autophagic bodies in vacuoles under starvation conditions in the absence of vacuolar proteinases (Tsukada and Ohsumi, 1993). Almost simultaneously, Thumm et al. isolated several *aut* mutants of *S. cerevisiae* using the same method as Ohsumi et al. and identified aut1, which encodes 310 amino acids (Thumm et al., 1994; Schlumpberger et al., 1997). In 2000, a sequence analysis suggested that *apg3* is identical to *aut1* (both named *Atg3* for unified), and its gene product is an E2-like enzyme in the Atg8 lipidation system and the cytoplasm to vacuole targeting (Cvt) pathway (Ichimura et al., 2000; Kim et al., 2001).

During autophagosome elongation, the LC3 precursor is cleaved by ATG4B following exposure of Gly120 to form LC3-I;, which is activated by the E1-like activating enzyme, ATG7. LC3-I; is subsequently transferred to the E2-like conjugated enzyme, ATG3, and finally links to PE with the help of the E3-like ligase ATG12–ATG5 conjugate (Tanida et al., 2002a, b, 2004; Hanada et al., 2007).

ATG3 contributes to autophagosome formation by interacting with ATG7, ATG8, ATG12, and the lipid membrane. A recent study has shown that human ATG3 induces membrane aggregation *in vitro*, indicating that ATG3 contributes to vesicle restraint preceding fusion events in autophagosome elongation, which means that the function of ATG3 in autophagosome biogenesis may be more complex (Hervas et al., 2017). ATG3 is also required for Atg8/LC3 lipidation, not only for canonical autophagy but also for noncanonical LC3 lipidation (Chang et al., 2013). Translation of ATG3 also affects autophagy in mammals, *Caenorhabditis elegans*, and yeast. As reported recently, eukaryotic translation initiation factor 5A (eIF5A) is required for LC3 lipidation by assisting the ribosome via its hypusine residue to increase the translation of ATG3 at its DDG motif, which is a motif conserved in eukaryon and displays eIF5A hyperdependency. The connection of eIF5A with ribosomes is enhanced when autophagy is induced (Lubas et al., 2018). Hence, ATG3 is a key autophagy molecule worthy of further studies.

### Structure of ATG3

**FIGURE 1:**
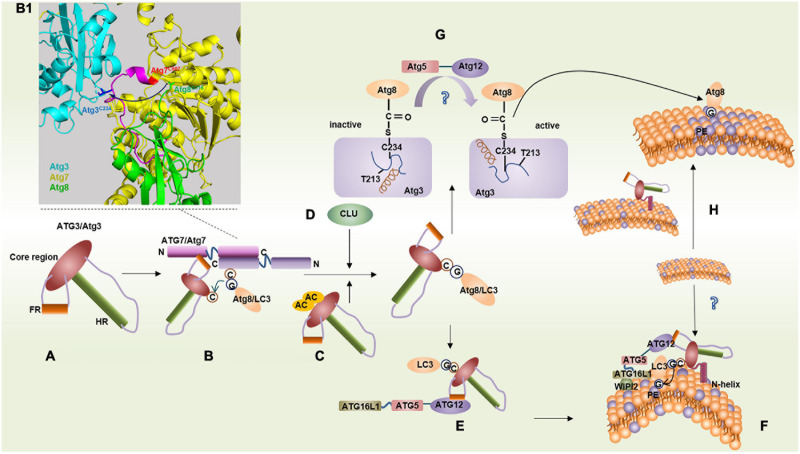
Binding features of ATG3/Atg3 in two conjugation systems. **(A)** Structure of ATG3/Atg3. **(B)** LC3/Atg8 transfers from ATG7/Atg7 onto ATG3/Atg3 via a transmechanism. **(B1)** Model representation of the Atg7∼Atg3∼Atg8 complex by aligning Atg7–Atg3 (PDB ID: 4GSL) with the Atg7^*CTD*^–Atg8 (PDB ID: 3RUI). Atg8^*G*116^ transfers from Atg7^*C*507^ to Atg3^*C*234^, where Atg3 binds with another Atg7 within an Atg7 dimer. **(C)** Acetylation of K19 and K48 of Atg3 promotes Atg3–Atg8 conjugate. **(D)** Molecular chaperone clusterin (CLU) promotes ATG3-LC3 heterocomplex stability. **(E)** ATG3^*FR*^ binds with ATG12 conjugate facilitating LC3 lipidation. **(F)** ATG3 N-helix inserts highly curved membrane mediating LC3 lipidation. **(G)** In yeast, the interaction between the loop (blue) containing the catalytic Cys234 and helix G (brown) in Atg3 makes Cys234 face away from Thr213, which suppresses Atg3 conjugase activity. Atg12–Atg5 leads to the reorientation of Cys234 by some unknown means, and then Cys234 faces to Thr213 to facilitate the Atg8 lipidation. **(H)** N-terminal domain of Atg3 can increase local PE density on the membrane.

Atg3/ATG3 is a dynamic protein lacking a rigid structure. In fact, approximately one-third of the Atg3/ATG3 sequences are missing in the crystal structure (Popelka et al., 2014). The structure of Atg3/ATG3 resembles a hammer composed of a head region (core region) and a handle region (HR). The core region with an α/β-fold is topologically similar to canonical E2 enzymes, such as ubiquitin-conjugating enzyme 9 (Ubc9), although they have little sequence homology with each other. The HR consists of a long α-helix and a partially disordered loop region. Furthermore, there is a “floating” helix C called FR at the interface between the core region and HR (Figure 1A; Yamada et al., 2007).

### Binding Features of ATG3

#### Interaction Between ATG3 and ATG7

ATG3 forms an E1–E2 complex with ATG7, which is unique compared with other protein-conjugation systems. An *in vitro* pull-down assay showed that Atg3^*HR*^ and Atg3^*FR*^ are responsible for binding with Atg8 and Atg7, respectively (Tanida et al., 2002a; Yamada et al., 2007).

In yeast, a short α-helix of Atg3^*FR*^ can insert a hydrophobic groove in the N-terminal domain (NTD) of Atg7, in which the Atg3 residue, Met139, docks in a hydrophobic pocket formed by the Atg7 residues, Phe93, Lys94, Trp139, and Pro283 (Yamaguchi et al., 2012). Thereafter, Atg8 transfers from Atg7 to Atg3 and forms a thioester bond with Cys234 of Atg3 via a transmechanism. Atg7 can form a dimer by interacting with two active-site cysteine residues in the C-terminal domain (CTD). The Atg8 thioester linked to the CTD of one Atg7 transferred to Atg3 interacted with the NTD of the other Atg7 within a dimer (Figures 1B,B1; Komatsu et al., 2001; Hanada et al., 2007; Taherbhoy et al., 2011). A similar pattern of interaction between ATG3 and ATG7 was also found in mammals, such as mice. However, in plants such as *Arabidopsis thaliana*, AtATG3^*CORE*^ binds to the C-terminal side of AtATG7^*NTD*^ to form an L-shaped structure through hydrophobic interactions (Yamaguchi et al., 2012).

#### Interaction Between ATG3 and LC3/ATG8

LC3/Atg8 usually binds to the catalytic active sites of ATG3/Atg3 via a thioester bond (Yamada et al., 2007). In mammals, the molecular chaperone clusterin (CLU) can promote ATG3-LC3 heterocomplex stability and LC3 lipidation via direct interaction with the LC3 protein, and the ATG3-LC3 complex is significantly reduced in CLU-silent cells (Figure 1D; Zhang et al., 2014). In yeast, Atg3 is a substrate of the histone acetyltransferase Esa1, and acetylation of Lys19 and Lys48 of Atg3 positively regulates autophagy by promoting Atg3–Atg8 and Atg8–PE conjugates (Figure 1C; Hanada et al., 2009).

Atg3 can also bind to Atg8 in the absence of a thioester bond in yeast (Yamada et al., 2007). Nuclear magnetic resonance spectroscopy revealed that Atg3 directly interacts with Atg8 through the WEDL (Trp^270^–Glu^271^–Asp^272^–Leu^273^, called Atg3^*AIM*^) sequence conserved in eukaryotes in HR. Atg3^*AIM*^ affects the Cvt pathway and the formation of Atg8–PE but does not affect the formation of the Atg8–Atg3 thioester intermediate (Yamaguchi et al., 2010). Interestingly, Atg3^*AIM*^ in *Toxoplasma gondii* contains an FADI (Phe–Ala–Asp–Ile) sequence and a WLLP (Trp–Leu–Leu–Pro) sequence, which is different from that of yeast Atg3^*AIM*^ (Chen et al., 2016; Liu et al., 2018; Varberg et al., 2018). Such findings indicate that Atg3^*AIM*^ may be species specific; thus, antitoxoplasmosis drugs and anti-*Plasmodium* drugs that target Atg8–Atg3 could be developed.

#### Interaction Between ATG3 and ATG12

In *Atg3^–/–^* MEFs, the Atg12–Atg5 complex is markedly reduced. In contrast, the overexpression of ATG3 could facilitate the formation of the ATG12–ATG5 complex in humans (Tanida et al., 2002b; Sou et al., 2008). The yeast Atg12–Atg5 conjugate is unnecessary for Atg8–PE conjugation *in vitro* at pH 7.58 but is indispensable *in vivo* (Suzuki et al., 2001; Yamada et al., 2007). In canonical E2 enzymes, such as Ubc9, an asparagine residue promotes ubiquitin or ubiquitin-like proteins linked to E2 enzymes through a thioester bond to transfer to a lysine residue of the substrate (Wu et al., 2003; Yunus and Lima, 2006). However, in Atg3, the corresponding amino acid is a threonine residue that is conserved in all known homologs (Thr213 in *S. cerevisiae*), and its Atg3 catalytic center is rearranged, with Cys234 reoriented toward Thr213 in the presence of Atg12–Atg5; this reorientation of Cys234 enhances the conjugate activity of Atg3 (Figure 1G; Sakoh-Nakatogawa et al., 2013). Hence, the Atg12–Atg5 conjugate is regarded as an E3-like ligase in the Atg8 lipidation system. However, the interaction between Atg3 and Atg12 has not been reported in yeast, and how Atg12–Atg5 plays an E3-like ligase role remains unclear.

In mammals such as humans, ATG3 can communicate with the E3-like ligases, ATG12–ATG5 to ATG16L1, by forming a β-sheet between ATG3^*FR*^ and ATG12. A 13-residue-long sequence of ATG3^*FR*^ called ATG3^*RIA12*^, which is highly conserved in eukaryotes except in fungi, is responsible for binding to ATG12 (Figure 1E; Metlagel et al., 2013). Therefore, in yeast, Atg12–Atg5 might act as an E3-like enzyme indirectly because there may not be a binding site for Atg12 in Atg3 based on the current evidence.

ATG3 can also interact with free ATG12 more preferentially than the ATG12–ATG5 conjugate, and the excess interaction of ATG3 with free ATG12 could partially inhibit the interaction between ATG7 and free ATG12, resulting in the suppression of LC3 lipidation (Tanida et al., 2002a), likely for the reason that the linear sequence of human ATG3^*FR*^ that binds to ATG12 and ATG7 is overlapped at the 157–176 amino acids (Qiu et al., 2013; Ohashi and Otomo, 2015).

#### Membrane Binding and Sensitivity of ATG3

The N-terminal of Atg3 was found to be essential for binding with PE in Atg8 lipidation according to a flotation assay. However, the 1–20 amino acids within the N-terminal cannot function alone, suggesting that other regions of Atg3 are required for the interaction with PE-containing liposomes. The researchers hypothesized that Atg8 moves from Cys234 to the N-terminal of Atg3 and then combines with PE (Hanada et al., 2009). In mice and humans, the 20 N-terminal amino acids of ATG3 form an amphipathic helix that is sensitive to a highly curved membrane when the membrane has a low PE density (<30 mol%) and acts via membrane insertion (Figure 1F; Dancourt and Melia, 2014; Nath et al., 2014). Recently, a study suggested that human ATG3 communicates information from the N-terminal amphipathic helix to the C-terminus to catalyze LC3–PE conjugation (Ye et al., 2021). Under these conditions, LC3 lipidation can be made very efficient even in the absence of the ATG12–ATG5∼ATG16L1 complex *in vitro*; however, *in vivo*, the location of ATG3-mediated lipidation depends on the localization of ATG16L1 (Figure 1F; Fujita et al., 2008; Dancourt and Melia, 2014; Nath et al., 2014). The N-terminal–positive residues, Lys9 and Lys11, are essential for recognizing phospholipid-negative moieties, and the presence of anionic phospholipids, particularly anionic lipids with a negative intrinsic curvature, can suppress the preference for highly curved membranes (Hervas et al., 2017). Acetylation of ATG3 Lys19/Lys48 can also enhance its binding to PE-containing liposomes and the endoplasmic reticulum (ER), thereby promoting the lipidation process (Li et al., 2017).

Interestingly, PE density under physiological conditions ranges from 15 to 20 mol% in yeast ER and Golgi (Nair et al., 2011), which have been implicated as possible sources of the autophagosome membrane. However, whether ATG3 highly curved membrane sensing is predominant *in vivo* and how the highly curved membrane is formed remain unknown. A recent study found that the NTD of Atg3 not only serves as a membrane anchor but also attenuates lateral diffusion of PE, thereby increasing the local PE density on the membranes. Atg8 lipidation can thus bypass the Atg3 highly curved membrane sensing and conjugate to PE (Figure 1H; Wang et al., 2020). However, considering the assay was performed *in vitro*, whether Atg3 can also increase local PE density on the membranes *in vivo* remains unknown. Another study detected highly curved membrane formation in synapses, where the phosphorylation of the endophilin A–BAR domain by leucine-rich repeat kinase 2 served as a docking station of ATG3 and facilitated highly curved membrane formation (Soukup et al., 2016).

Nevertheless, three fundamental aspects of membrane binding and the sensitivity of ATG3 remain unclear: Can ATG3 induce a curved membrane by itself? Are proteins that can induce curved membranes, such endophilin A, present at the isolation membranes in other tissues? And how does ATG3 interact with other proteins on the isolation membrane? Further studies will provide more details to enable a better understanding of the molecular mechanism.

#### Switch Mechanism of ATG3

Recently, an *in vitro* assay of yeast ATG proteins identified a region called Atg3^*E*123I*R*^ (Ile129-Lys142) that could autoinhibit the catalytic Cys234 residue of Atg3 within Atg3^*FR*^. Atg3^*E*123I*R*^ is relocated when binding to Atg7^*NTD*^. Thereafter, the Atg3 catalytic core is rearranged, leading to activated Cys234 that attacks the Atg7–Atg8 intermediate, forming the Atg3–Atg8 thioester intermediate. With Atg7 leaving, Atg3^*E*123I*R*^ autoinhibits the catalytic Cys234 residue again to keep the Atg3–Atg8 intermediate stable. When Atg12–Atg5∼Atg16 binds to Atg3^*E*123I*R*^, the Atg3–Atg8 intermediate participates in a nucleophilic attack and Atg8–PE formation (Zheng et al., 2019).

In mammals and yeast, the catalytic cysteine residue of ATG3/Atg3 is bound to LC3/Atg8 through a stable thioester when autophagy is inactive. However, the thioester becomes transient upon autophagy stimulation, followed by the exposure of catalytic thiols, and then forms a disulfide heterodimer with ATG7/Atg7 or a glutathione adduct. This process is upregulated, which might contribute to impaired autophagy during aging, partly in aged mouse tissues (Burgoyne, 2018).

## Regulation Factors and Pathophysiological Roles of ATG3

Autophagy is involved in a wide range of physiological processes; for example, ATG3 can inhibit autophagy-induced apoptosis in inactivated Sendai virus (HVJ-E)–treated cells (Wang T. et al., 2018). The downregulation of mouse Atg3 expression results in compromised embryonic stem cell self-renewal, pluripotency, and differentiation (Sou et al., 2008). Its multiple physical functions are realized through a crosstalk with diverse cellular pathways via interactions among pivotal gene components. ATG3 is a key component of autophagy and is required to keep mammals alive (Sou et al., 2008). The role of ATG3 in autophagy-mediated physical functions can be regulated by its binding partners at different levels (Table 1).

**TABLE 1:** ATG3 modulators and functions.

**Modulated types**	**Modulators**	**Functions**	**References**
**Transcription level**			
	GATA-1	Induces ATG3 in erythroleukemia	Harr et al., 2011
	LAPTM4B	Activates ATG3 in HCC	Wang et al., 2019
	GR	Increases ATG3 in folic acid deprivation cells	Sun et al., 2016
**Translation level**			
	MiR-16	Upregulates ATG3 in NSCLC by negative regulation	Wang H. et al., 2018
	MiR-204-5P	Reduces ATG3 in NSCLC	Kang et al., 2019
	MiR-1	Reduces ATG3 in NSCLC	Hua et al., 2018
	MiR-365	Upregulates ATG3 in HCC	Yang et al., 2019
	MiR-204	Upregulates ATG3 in HCC	Li et al., 2020
	MiR-431-5p	Upregulates ATG3 in colon cancer by negative regulation	Huang et al., 2019
	MiR-155	Downregulates ATG3 in tuberculosis	Etna et al., 2018
	HDAC1	Downregulates ATG3	Du et al., 2019
	MiR-495	Downregulates ATG3	Li et al., 2016
	MiR-23a	Downregulates ATG3	Li et al., 2018
	MiR-206	Downregulates ATG3	Kong et al., 2019
**Posttranslational modification**			
	Acetyltransferases	Promotes cancer cell survival	Tan et al., 2016
	FLIPs	Suppresses autophagy	Lee et al., 2009
	Caspase-3	Cleaves ATG3	Norman et al., 2010
	Caspase-6	Cleaves ATG3	Norman et al., 2010
	Caspase-8	Cleaves ATG3	Norman et al., 2010; Oral et al., 2012
	TNFAIP8	Creates cellular autophagy	Niture et al., 2020
	PDCD6IP	Distributes late endosome	Murrow et al., 2015
	Calpain 1	Cleaves ATG3	Norman et al., 2010
	Calpain 2	Cleaves ATG3	Zhao et al., 2016
	Beclin-1	Protects liver	Wang et al., 2011
	GAPCs	Regulates autophagy negatively	Han et al., 2015; Liu T. et al., 2017; Ismayil et al., 2020
	Hat1	Appressorium formation and pathogenicity	Yin et al., 2019

*GATA-1, a hematopoietic transcription factor; LAPTM4B, lysosomal-associated protein transmembrane-4 beta; GR, glucocorticoid receptor; MiR, microRNA; HCC, hepatic cellular cancer; HDAC1, histone deacetylase 1; FLIP, FLICE-like inhibitor protein; NSCLC, non–small cell lung cancer; TNFAIP8, tumor necrosis factor α–induced protein 8; PDCD6IP, ESCRT-associated protein Alix; GAPCs, glyceraldehyde-3-phosphate dehydrogenases; Hat, histone acetyltransferase.*

### ATG3 in Cancer

**FIGURE 2:**
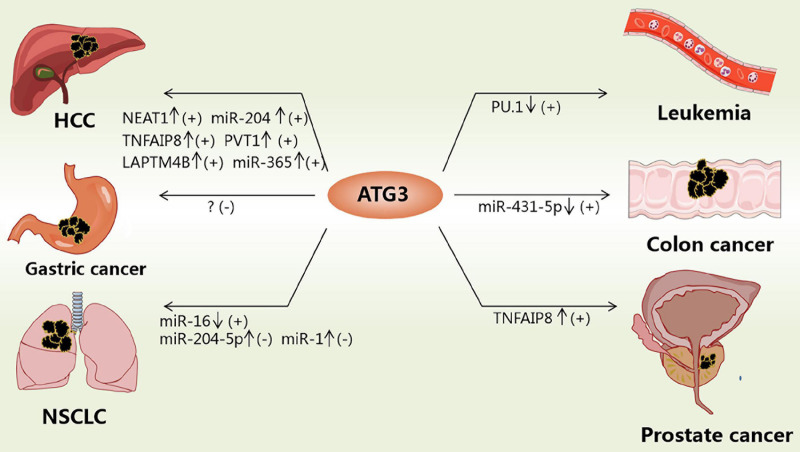
Functions of ATG3 in cancers. ATG3 could be modulated by different factors in cancers. “↓” means the corresponding factor is downregulated; “↑” means the corresponding factor is upregulated; “+” means inducing cancers; “-” means suppressing cancers.

The expression of ATG3 changes significantly in various types of cancer tissues (Table 2), indicating that the expression level of ATG3 is closely related to cancer. For example, *ATG3* knockdown remarkably suppressed the proliferation and invasion of colon cancer cells (Huang et al., 2019). In addition, ATG3 can be modified by acetyltransferases recruited by Myc box II, a region within Myc-nick, which is a cleavage product of Myc being present in most tumor samples, leading to the upregulation of autophagy and cancer cell survival (Conacci-Sorrell et al., 2014). ATG3 can also interact with cellular and viral FLIPs (death effector domains) to suppress autophagy, resulting in tumor development (Lee et al., 2009). In general, the expression and modification of ATG3 play important roles in tumor development and progression (Figure 2).

**TABLE 2:** Changes of ATG3 in different types of tumors.

**Tumor types**	**ATG3**	**References**
Myelodysplastic syndrome	↓	Ma et al., 2013; Wang et al., 2014
Myeloid leukemia	↓	Ma et al., 2013
Erythroleukemia (JAK2 V617F mutation)	↑	Harr et al., 2011
Non–small cell lung cancer	↑	Hua et al., 2018; Wang H. et al., 2018
Hepatic cellular cancer	↑	Wang et al., 2019; Li et al., 2020
Gastric cancer tissues	↑	Cao et al., 2016
Colon cancer	↑	Huang et al., 2019
Prostate cancer	—	Niture et al., 2018

*“—”, not determined.*

A gene expression microarray study revealed that ATG3 was downregulated in myelodysplastic syndrome patients and patients progressing to leukemia (Ma et al., 2013; Wang et al., 2014). However, hematopoietic cells expressing BCR-AbI (a constitutively active oncogenic kinase) are highly sensitive to autophagy and fail to generate leukemia cells without ATG3 (Altman et al., 2011). PU.1 is a positive transcriptional regulator of *ATG3*, and low expression of PU.1 may account for the low expression of ATG3 in acute myeloid leukemia (Jin et al., 2018). For example, the overexpression of ATG3 in SKM-1 cells can induce autophagy and increase sensitivity to bortezomib treatment (Zhuang et al., 2016). However, ATG3 in human erythroleukemia cells with JAK2 V617F mutation is induced, whereby JAK2 V617F inactivates protein arginine methyltransferase 5 and promotes GATA-1 to bind with the ATG3 promoter (Harr et al., 2011). Such findings suggest that the low expression of ATG3 is essential for maintaining leukemia, and restoring autophagic activity might be beneficial in differentiation therapies. Hence, ATG3 could be a potential target for treating leukemia by increasing its expression or specifically inhibiting its degradation if specific mutations can be excluded.

In non-small cell lung cancer (NSCLC) patients, the expression level of miR-16 was significantly downregulated, whereas that of ATG3 was upregulated, with the 3’-UTR of ATG3 as the direct target of miR-16 (Wang H. et al., 2018). MiR-204-5p binds to the 3’-UTR of ATG3 to inhibit the expression of ATG3. ATG3 overexpression could reverse the effect of miR-204-5p on NSCLC cell proliferation inhibition (Kang et al., 2019). MiR-1 overexpression could improve the *cis*-platinum sensitivity of NSCLC cells by reducing the expression of ATG3, causing impaired ATG3-mediated autophagy, which provides a potential target for relieving antitumor drug resistance (Hua et al., 2018). In summary, ATG3 in patients with NSCLC is upregulated and protects NSCLS cells, such as A549, through autophagy. Hence, drugs that target the degradation of ATG3 or the reduced expression of ATG3 may be designed to suppress NSCLC cell proliferation.

ATG3 mRNA expression levels were found to be higher in hepatic cellular cancer (HCC) tissues than in adjacent nontumor liver tissues. The transcription of *ATG3* is activated by lysosomal-associated protein transmembrane-4β (LAPTM4B) to modulate apoptosis and autophagy in HCC cells (Wang et al., 2019). Moreover, long noncoding RNA (lncRNA) plasmacytoma variant translocation 1 expression is increased, which can facilitate autophagy by sponging miR-365 to target the 3’-UTR of ATG3 and upregulate ATG3 (Yang et al., 2019). Tumor necrosis factor α–induced protein 8 (TNFAIP8) can interact with ATG3 and subsequently create cellular autophagy events that promote cell survival and drug resistance (Kristensen et al., 2012; Niture et al., 2018, 2020). The Cancer Genome Atlas showed that the lncRNA nuclear enriched abundant transcript 1 is upregulated in HCC tissue and promotes HCC autophagy and sorafenib resistance by sponging miR-204 to upregulate ATG3 expression (Li et al., 2020). Some studies found that the ectopic expression of CD147-ICD causes the accumulation of ATG3 via the nuclear factor κB–TRAIL–caspase8–ATG3 axis, which increases the viability of cisplatin-treated HCC cells by enhancing autophagy in HCC cells (Wu et al., 2017). The above studies indicate that drug resistance is associated with high expression of ATG3 to some extent. In addition, the overexpression of ATG3 in HCC increases autophagic flux, which is beneficial for the growth of HCC cells. Therefore, downregulation of ATG3 or destruction of E2 enzyme activity while using drugs such as sorafenib to treat HCC could improve drug sensitivity.

ATG3 is upregulated in many other tumors. In gastric cancer tissues, a study showed that the expression of ATG3 is upregulated and acts as a favorable independent prognostic factor, as supported by overall survival analysis (Cao et al., 2016). However, another study showed that in colon cancer tissues, the expression of ATG3 was upregulated. As a result, ATG3 could promote proliferation and invasion in colon cancer, whereas the downregulation of ATG3 could suppress the progression of colon cancer (Huang et al., 2019). In prostate cancer cells, TNFAIP8 can interact with ATG3 and subsequently create cellular autophagy events that promote cell survival and drug resistance (Kristensen et al., 2012; Niture et al., 2018, 2020), thereby indicating that ATG3 may play different roles in different cancers.

### ATG3 in Ischemia–Reperfusion Injury

ATG3 expression contributes to ischemia–reperfusion (I/R) injury. A recent study found that the upregulation of mitochondrial RNA processing endoribonuclease and the upregulation of ATG3 caused by the downregulation of miR-206 might worsen myocardial I/R injury (Kong et al., 2019). However, in fatty livers, ATG3 seems to be a protective factor against I/R injury. Another study revealed that, compared with normal livers, fatty livers are more susceptible to I/R injury because of the higher expression of calpain 2 after I/R, and amino acids 92–97 of ATG3 can be cleaved by calpain 2, causing the inhibition of autophagy. Furthermore, the *in vitro*/*in vivo* overexpression of ATG3 could enhance autophagy and reduce cell death after I/R injury in fatty liver (Zhao et al., 2016). However, the interaction between ATG3 and beclin-1 could protect the livers of old mice from I/R injury to some extent (Wang et al., 2011). Currently, the specific mechanism remains unaddressed because ATG3 is downstream of beclin-1. Based on the evidence, the effect of ATG3 on I/R appeared to differ in different organs. For example, ATG3 plays a negative role in myocardial I/R injury but plays a protective role in I/R injury in fatty livers.

### ATG3 in the Clearance of Pathogens

ATG3 can aid in the clearance of pathogens by regulating autophagy. A study found that live and virulent *Mycobacterium tuberculosis* (Mtb) significantly stimulated the expression of miR-155 in dendritic cells. MiR-155 binds to the ATG3 3’-UTR to inhibit the translation of ATG3, causing autophagy inhibition and Mtb survival (Etna et al., 2018). In infected cells, the expression of ATG3 is required for immunity-related GTPase (IRG) to dock to *T. gondii* and *Chlamydia trachomatis* pathogen-containing vacuoles, possibly by activating IRG, thereby protecting host cells (Haldar et al., 2014). Hence, developing novel drugs that would boost autophagy is a new therapeutic strategy against tuberculosis, or drugs targeting miR/lncR to promote ATG3 expression might be an underlying tool to clear pathogens.

### ATG3 in Organelle Homeostasis

ATG3-dependent autophagy is critical for mitochondrial homeostasis (Liu et al., 2016). A previous study showed that ATG3-depleted *T. gondii* exhibited remarkable mitochondrial fragmentation (Besteiro et al., 2011). Dysfunctional mitochondria accumulated in adipocytes lacking ATG3 due to postdevelopmental impairment of autophagy, resulting in increased lipid peroxidation, adipose tissue inflammation, systemic insulin resistance, and Nrf2 and keap1 activation (Cai et al., 2018). This accumulation of damaged mitochondria may be owing to mitophagy defects in the absence of ATG3.

**FIGURE 3:**
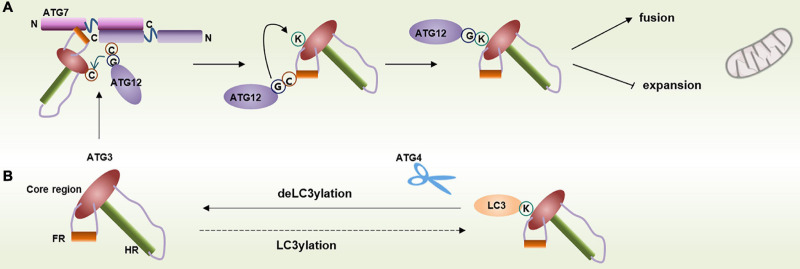
Modification of ATG3 residue Lys243. **(A)** Lys243 within ATG3 can conjugate with free ATG12 and contribute to mitochondrial homeostasis. **(B)** Lys243 within ATG3 can also form a conjugate with LC3 via a process called LC3ylation, and ATG4 can cleave the conjugate.

ATG3 can form an ATG12–ATG3 complex with unconjugated ATG12 in mammals. The ATG12–ATG3 conjugate is produced by the autocatalytic reaction of ATG3, where the ATG12 thioester linked to the catalytic cysteine of ATG3 is transferred to the lysine residue (Lys243 of human ATG3) of the same ATG3 molecule (Tanida et al., 2002a; Radoshevich et al., 2010). The ATG12–ATG3 complex is responsible for mitochondrial homeostasis, and cells lacking ATG12–ATG3 undergo mitochondrial mass expansion and mitophagy blocking (Figure 3A; Radoshevich et al., 2010). Interestingly, a recent study has reported a new posttranslational modification of LC3, named LC3ylation. In this modification, LC3 can also conjugate to the ATG3 residue, Lys243, to form an LC3–ATG3 conjugate. The LC3–ATG3 conjugate can be cleaved by ATG4B, which is defined as deLC3ylation (Figure 3B; Agrotis et al., 2019). However, the specific mechanism and physiological function of LC3ylation should be further studied.

ATG3 plays a key role in late endosome function. In fact, ATG12–ATG3 is essential for multitudinous PDCD6IP-mediated functions, including late endosome distribution, exosome biogenesis, and viral budding (Murrow et al., 2015). PDCD6IP contains three structural domains, including an N-terminal Bro1 domain, a C-terminal proline-rich domain (PRD), and the V domain binding to the YPXnL motifs (Strack et al., 2003; Baietti et al., 2012). The YPXnL-binding site in the V domain is inhibited by intramolecular interaction of the PRD with the Bro1 and V domains. ATG12–ATG3 binds to the Bro1 and V domains to release the YPXnL-binding site and then supports multiple PDCD6IP functions (Murrow et al., 2015).

### ATG3 in Plant Growth and Plant-Associated Microbiome

In *Nicotiana benthamiana* plants, ATG3 interacts with cytosolic glyceraldehyde-3-phosphate dehydrogenases (GAPCs) to negatively regulate autophagy (Han et al., 2015). In potato, the interaction of StATG3 and StGAPCs might contribute to the maintenance of tuber apical dominance, possibly by preventing cell death in the tuber apical bud meristem (Liu T. et al., 2017). Such finding suggests that the interaction between ATG3 and GAPCs is important for the growth of plants, and chemicals targeting ATG3-GAPCs could be designed and used to maintain or eliminate tuber apical dominance to improve production or control plant type.

ATG3 can relieve viral symptoms via autophagy in plants. ATG3 is a regulator of plant immunity-related cell death that limits the extent of spread of tobacco mosaic virus (TMV)–induced hypersensitive response programmed cell death (Liu Y. et al., 2005). As a result, ATG3 could protect plants from TMV infection, likely through autophagy. In cotton leaf curl Multan betasatellite, CLCuMuB βC1 can activate autophagy by disrupting GAPCs–ATG3 interactions, and mutant virus carrying βC13A, which could not induce autophagy, showed more severe viral symptoms and viral DNA accumulation (Ismayil et al., 2020). However, the acetylation of pathogenic ATG3 seemed to be an adverse factor for plants because of the activation of autophagy in pathogens. In the rice blast fungus, *Magnaporthe oryzae*, ATG3 is acetylated by Hat1 to facilitate autophagy and function in appressorium formation and pathogenicity (Yin et al., 2019). These studies suggest that autophagy in plants is facilitated or suppressed in pathogens by modulating ATG3 to alleviate viral infection.

## Autophagy-Independent Functions of ATG3

As a crucial gene of autophagy, several autophagy-related functions of *ATG3* have been reported; however, only a few studies have investigated its autophagy-independent functions.

ATG3 is not only a key component of autophagy but also functions in DNA damage–induced mitosis in an autophagy-independent manner. Research has shown that ATG3 is degraded via Tyr203 phosphorylation during etoposide or cisplatin treatment, causing DNA damage in cancer cell lines. However, the inhibition of ATG3 degradation during DNA damage could facilitate DNA damage–induced mitotic catastrophe, potentially via the interaction between ATG3 and BAG3 (BCL2-associated athanogene 3), which is a key protein in the mitotic process, even in the absence of ATG7 (Ma et al., 2017).

In *Fusarium oxysporum*, each wild-type hypha contains only one nucleus in one hyphal compartment; however, the *Foatg3* null mutant hyphae were found to contain more than one nucleus in one hyphal compartment, causing virulence reduction, which indicated that Foatg3 is a crucial target in the treatment of root crop dry rot disease (Liu T. et al., 2017; Khalid et al., 2019). In addition, unlike *Atg16^–/–^* (another essential gene in LC3 lipidation system) mice that can survive, mice without Atg3 died within 1 day of birth. *Atg3^–/–^* MEFs could also be subcultured, indicating that Atg3 is important for cell differentiation (Saitoh et al., 2008; Sou et al., 2008; Liu T. et al., 2017).

ATG3 might play important roles in cell differentiation and mitosis in an autophagy-independent manner. Although only limited evidence of the nonautophagic roles of ATG3 has been reported thus far, we believe that more autophagy-independent functions may be discovered through future investigations.

## Conclusion and Perspectives

Autophagy is crucial for maintaining cellular homeostasis and plays an essential role in infectious diseases, cancers, and neurodegenerative diseases (Choi et al., 2013). As a core autophagy-related protein, ATG3 is necessary for LC3 lipidation via its E2-like–conjugated enzyme and membrane-binding functions; however, details such as the source of highly curved membranes *in vivo*, mechanism of increased local PE density by Atg3, and the mode of Atg5–Atg12 required to affect Atg8 lipidation in yeast remain unknown. As a result, the specific mechanism requires further investigation. ATG3 can form an ATG3–ATG12 conjugate at Lys243, which is crucial for maintaining mitochondrial homeostasis (Radoshevich et al., 2010). Interestingly, a recent study has reported that LC3B conjugation to ATG3, called LC3ylation, also occurs at Lys243 in the absence of ATG4. However, whether there are new physiological functions modulated by this conjugate remains unknown, thereby warranting further studies (Agrotis et al., 2019).

ATG3 plays a key role in all cancer types. In established solid tumors, such as NSCLC, ATG3 is significantly upregulated, resulting in increased autophagy flux, which may confer a survival advantage for solid tumor cells (Harr et al., 2011; Sun et al., 2016; Kang et al., 2019; Yang et al., 2019; Li et al., 2020). Thus, inhibitors that can downregulate ATG3 may be developed as antisolid tumor drugs for most tumors, except for gastric cancer; this is because ATG3 is a favorable independent prognostic factor of this cancer type (Cao et al., 2016). However, ATG3 is downregulated in blood tumors, such as leukemia without the JAK2 V617F mutation (Lee et al., 2009; Wang et al., 2014). Hypoxia upregulated oriental river prawn MnATG3 mRNA expression in a time-dependent manner, suggesting that autophagy could protect crustaceans from hypoxia (Sun et al., 2019). Therefore, ATG3 downregulation in blood cancer is likely because oxygen is abundant in blood vessels, which increases the proliferation rate of tumor cells. Autophagy did not occur in this process because ATG3 might regulate blood tumors in an autophagy-independent manner. Overall, ATG3 regulators could be developed to modulate tumor progression; however, the lack of an effective assay to detect ATG3 activity is a current limitation that should be addressed in a further study.

Recently, researchers have found several autophagy-independent functions of ATG3, including its promotion of DNA damage–induced mitotic catastrophe (Liu Y. et al., 2005; Ma et al., 2017; Khalid et al., 2019). Such findings suggest that the functions of ATG3 are more complex. Owing to the functions of ATG3 in parasites and plants, it could be a potential target for protecting hosts from parasites and increasing crop yields. We anticipate the discovery of more functions of ATG3 in the future. Furthermore, we believe that the novel posttranslational modifications of ATG3 might be a good focal point for excavating its new functions.

## Author Contributions

DF conceived the review, consulted the literature, and drafted the figures and the manuscript. HX, TH, and HS consulted the literature and helped with the drawing and manuscript revision. ML came up with the idea and wrote the final paper with the feedback from all authors.

## Conflict of Interest

The authors declare that the research was conducted in the absence of any commercial or financial relationships that could be construed as a potential conflict of interest.

## Abbreviations

Asp: aspartic acid
ATG/Atg: autophagy-related gene
Ala: alanine
BAG3: BCL2 associated athanogene 3
BLAST: Basic Local Alignment Search Tool
CTD: C-terminal domain
CLU: clusterin
Cvt: cytoplasm to vacuole targeting
Cys: cysteine
DC: dendritic cells
eIF5A: eukaryotic translation initiation factor 5A
ER: endoplasmic reticulum
FR: “floating” helix C
FLIP: FLICE-like inhibitor protein
FLIPs: death effector domains
GATA-1: a hematopoietic transcription factor
GAPCs: glyceraldehyde-3-phosphate dehydrogenases
GS: glutathione
Glu: glutamic acid
Gly: glycine
GR: glucocorticoid receptor
Hat: histone acetyltransferase
HDAC1: histone deacetylase 1
HVJ-E: inactivated Sendai virus
HR: handle region
HCC: hepatic cellular cancer
I/R: ischemia–reperfusion
Ile: isoleucine
IRG: immunity-related GTPase
LAPTM4B: lysosomal-associated protein transmembrane 4 β
LC3: microtubule associated protein 1 light chain 3
LncR: long non-coding RNA
Lys: lysine
Leu: leucine
LRRK2: leucine-rich repeat kinase 2
MEFs: mouse embryo fibroblast cells
MiR: micoRNA
Mtb: *Mycobacterium tuberculosis*
NTD: N-terminal domain
NSCLC: non–small cell lung cancer
PE: phosphatidylethanolamine
PMT: posttranslational modification
PDCD6IP: ESCRT-associated protein Alix
Pro: proline
Thr: threonine
TGF- β 1: transforming growth factor β 1
TRAIL: TNF-related apoptosis-inducing ligand
TNF: tumor necrosis factor
TMV: tobacco mosaic virus
TNFAIP8: tumor necrosis factor α –induced protein 8
*T. gondii*: *Toxoplasma gondii*
Trp: tryptophan
Phe: phenylalanine
Ubc9: ubiquitin-conjugating enzyme 9.
